# Shear-wave elastography as a supplementary tool for axillary staging in patients undergoing breast cancer diagnosis

**DOI:** 10.1186/s13244-024-01747-z

**Published:** 2024-08-07

**Authors:** Riku Togawa, Fabian Riedel, Manuel Feisst, Sarah Fastner, Christina Gomez, André Hennigs, Juliane Nees, André Pfob, Benedikt Schäfgen, Anne Stieber, Markus Wallwiener, Jörg Heil, Michael Golatta

**Affiliations:** 1 Breast Unit, Department of Obstetrics and Gynecology, Heidelberg University Hospital, Heidelberg, Germany; 2 Institute of Medical Biometry (IMBI), Heidelberg University, Heidelberg, Germany; 3 Breast Unit, Sankt Elisabeth Hospital, Heidelberg, Germany; 4 Department of Diagnostic and Interventional Radiology, Heidelberg University Hospital, Heidelberg, Germany

**Keywords:** Breast cancer, Axillary staging, Ultrasound, Shear wave elastography

## Abstract

**Background:**

Preoperative evaluation of axillary lymph node status is crucial for the selection of both systemic and surgical treatment in early breast cancer. This study assessed the particular role of additional shear wave elastography (SWE) in axillary staging in patients undergoing initial breast cancer diagnostics.

**Methods:**

One hundred patients undergoing axillary lymph node biopsy due to a sonographically suspicious axillary lymph node were prospectively evaluated with SWE using virtual touch tissue imaging quantification (VTIQ). Mean values of tissue stiffness for axillary tissue and lymph node tissue were measured prior to core-cut biopsy of the lymph node. All lymph nodes were clip-marked during the biopsy. Cut-off values to differentiate between malignant and benign lymph nodes were defined using Youden’s index.

**Results:**

Lymph nodes with evidence of malignant tumor cells in the final pathological examination showed a significantly higher velocity as measured by SWE, with a mean velocity of 3.48 ± 1.58 m/s compared to 2.33 ± 0.62 m/s of benign lymph nodes (*p* < 0.0001). The statistically optimal cutoff to differentiate between malignant and benign lymph nodes was 2.66 m/s with a sensitivity of 69.8% and a specificity of 87.5%.

**Conclusions:**

Lymph node metastases assessed with SWE showed significantly higher elasticity values compared to benign lymph nodes. Thus, SWE provides an additional useful and quantifiable parameter for the sonographic assessment of suspicious axillary lymph nodes in the context of pre-therapeutic axillary staging in order to differentiate between benign and metastatic processes and support the guidance of definitive biopsy work-up.

**Critical relevance statement:**

Shear-wave elastography provides an additional useful and quantifiable parameter for the assessment of suspicious axillary lymph nodes in the context of pre-therapeutic axillary staging in order to differentiate between benign and metastatic processes and support guiding the definitive biopsy work-up.

**Key Points:**

- SWE is a quantifiable ultrasound parameter in breast cancer diagnosis.
- SWE shows a significantly higher velocity in malignant lymph nodes.
- SWE is useful in improving the sensitivity and specificity of axillary staging.

**Graphical Abstract:**

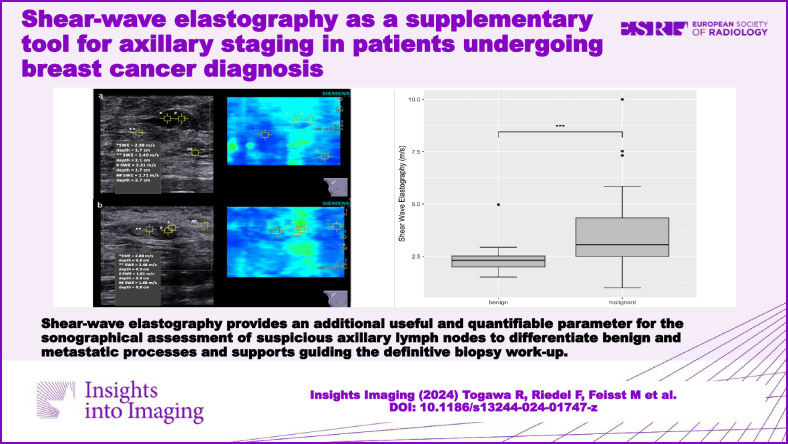

## Introduction

B-mode ultrasound of the axillary region plays a crucial role in the pre-therapeutic diagnostics of patients with early breast cancer [1, 2]. The status of axillary lymph nodes (LN) is a significant prognostic factor for disease recurrence and overall survival, influencing the selection of therapy regimens, including both systemic and surgical approaches [3, 4]. To reduce the risk of surgical overtreatment, axillary B-mode ultrasound must effectively identify patients with an unsuspicious axillary LN status and thus do not benefit from extensive axillary surgery [5]. Patients with involvement of axillary LNs who are not receiving neoadjuvant systemic therapy are recommended to undergo axillary lymph node dissection (ALND), whereas patients with negative nodal status undergo sentinel lymph node biopsy (SLNB) [1]. SLNB is associated with less morbidity such as lymphedema, seroma, and arm mobility impairment and sensitivity [6, 7]. According to national and international guidelines, axillary B-mode ultrasound is widely used in clinical routine [1]. Despite this fact, its diagnostic accuracy is still low and a standardization of LN positivity criteria is not present yet [8, 9].

Accurate axillary staging is also becoming increasingly important in the context of axillary operative de-escalation. In patients with LN involvement, instead of performing a complete removal of all lymph nodes in the axilla (ALND), surgeons may selectively target specific lymph nodes for removal under certain conditions, based on preoperative imaging or intraoperative assessment. This approach is becoming increasingly relevant, particularly as the response rate to neoadjuvant systemic therapy increases. By selectively targeting specific lymph nodes, this approach reduces the extent of surgery while still effectively addressing nodal staging [10–12].

Sonomorphological criteria for metastatic axillary LNs are eccentric cortical thickening, loss of fatty hilum, rounder shape, pathological color Doppler images such as increased, peripheral, and disruptive vascularity, and irregular margins [13–18]. These criteria possess a low positive predictive value, as the ultrasound signs are nonspecific and can also be observed in inflammation or reactively changed LNs. Although conventional B-mode ultrasound is widely used in clinical practice a consensus score on LN assessment has not been identified yet. The sensitivity and specificity of axillary staging using conventional B-mode ultrasound vary significantly across different studies, ranging between approximately 45–95% for each, respectively [4, 19]. Therefore, confirmation of suspicious LN in ultrasound by fine needle aspiration or core-cut biopsy is indispensable. These methods have a specificity of up to 100% but lower sensitivity ranging from 25% to 94% [20, 21]. For this reason, up to 20% of patients require a secondary ALND after SLNB [4, 22, 23].

Given this fact, additional tools are necessary to improve the diagnostic performance of axillary ultrasound. 2D shear wave elastography (SWE) measures tissue stiffness based on shear wave velocity and is an established complementary tool in diagnostics of the breast tissue since it has been integrated as a novel descriptor for malignancy in the BI-RADS 5th edition [24–26].

Increased tissue stiffness is a known predictor of malignancy. High stiffness is reported to be due to high levels of collagen, myofibroblasts, angiogenesis, inflammatory reaction, necrosis, and different tumor histologic biomarkers [27, 28].

It has been shown that SWE is also feasible in axillary LNs, but few studies have evaluated the diagnostic value of SWE in axillary staging [29–32]. In this single-center prospective diagnostic study, we assessed the potential of SWE to distinguish between benign and malignant axillary LNs in a clinical routine setting.

## Materials and methods

### Study design and enrollment

This is a single-center prospective diagnostic study. The study protocol was approved by the local ethics committee and additional written informed consent was obtained from each participating patient (S-396/2019). The study was conducted in a specialized diagnostic breast unit and included 100 consecutive patients with one or more suspicious axillary LNs and an indication for an axillary LN core-cut biopsy between June 2021 and December 2022. This cohort encompassed individuals attending the breast unit for various reasons, such as for routine surveillance following contralateral breast cancer treatment, those participating in routine breast cancer screening, as well as those seeking clarification of breast lesions. However, the study excluded patients diagnosed with malignancies other than breast cancer (e.g., lymphoma or sarcoma). Additional exclusion criteria comprised male sex, individuals younger than 18 years of age, inflammation of the breast, previous ipsilateral axillary surgery (i.e., history of SLNB or ALND), prior radiation therapy to the ipsilateral breast, or ongoing breast cancer treatment. All 100 LNs were evaluated using SWE prior to core needle biopsy. Each LN was clip-marked directly after the biopsy. SWE, as well as the biopsy, were performed by the same examiner. Two radiologists, as well as five gynecologists experienced in B-mode ultrasound, SWE, and biopsies, were eligible to include patients in this study.

### Conventional B-mode ultrasound and selection of the LNs

Patients were positioned identically for imaging with the ipsilateral arm in an elevated position. B-mode ultrasound was performed using Siemens Acuson S2000 or S3000 with a 9 MHz probe (Siemens Healthineers). Criteria for suspicious LNs in ultrasound were the presence of cortical hypertrophy > 3 mm, eccentric or focal cortical hypertrophy, round shape with complete or partial effacement of the fatty hilus, or expressing pathological color Doppler images [8, 33]. If one or more of these criteria were applicable to one or more axillary LNs, a core-cut biopsy was performed. The selection of the respective LN was up to the examiner who performed a B-mode ultrasound. Generally, the LN judged to be the most suspicious was selected for biopsy, although the location, as well as the relation to nearby vessels, may also influence LN selection. The long-axis and short-axis diameters of each suspicious LN were measured and documented. Biopsy was performed using 14G HistoCore® Automatic Biopsy System by BIP Biomed (Tuerkenfeld, Germany). Two or three samples were taken from each LN.

### SWE

SWE was performed using 2D-SWE systems Siemens Acuson S2000 or S3000 equipped with virtual touch tissue imaging quantification (VTIQ) software utilizing a 9 MHz probe (Siemens Healthineers). The VTIQ algorithm estimates the velocity of the induced shear waves which is correlated to tissue stiffness. SWE was always performed on the LN that was chosen for biopsy. The accuracy of the measurement was indicated by a quality map [34]. If the image was compromised due to compression or movement of the patient, the measurement had to be repeated.

SWE was performed with minimum compression induced by the transducer. Elasticity values were measured in meters per second (m/s), ranging from 0 to 10 m/s. Elasticity values from the regions of interest, namely the area with the highest velocity of the LN, as well as from the surrounding tissue were documented. The biopsy of the selected LN was performed in the same position. The previously selected LN was biopsied and marked with a clip. The biopsy of the LN was controlled with ultrasound. The clip was additionally documented by mammography in 96% of cases. In 80% of the cases, the clip could be seen on the mammogram; the other 20% could not be seen due to the clip’s localization.

### Pathological reference

Pathological examinations and immunohistochemistry (IHC) of the core-cut biopsies were conducted by the division head of gynecopathology, who possesses over 25 years of experience in breast pathologies, in accordance with national guidelines. These assessments were performed in a blinded setting, ensuring unbiased evaluation (i.e., pathologists were not aware of SWE findings) [35].

### Statistical analysis

This is an exploratory study. Statistical tests and resulting *p*-values can therefore only be interpreted descriptively. The study cohort was described by the measures of empirical distribution. Depending on the level of measurement, mean and standard deviation (SD), as well as absolute and relative frequencies were calculated. To compare the study cohort and sonomorphology of the LNs with respect to benign and malignant histopathology, an independent *t*-test and chi-square test were used. Receiver operating characteristic curves (ROC) were plotted to determine cutoff points yielding the maximal sum of sensitivity and specificity (Youden index). The area under the receiver operating characteristic curve (AUC) was additionally calculated. Statistical analysis was performed with R (version 4.1.0—© 2021, The R Foundation for Statistical Computing).

## Results

### Description of the study cohort

One hundred consecutive patients with suspicious axillary LNs in the ultrasound examination were enrolled. Five patients were excluded based on the exclusion criteria. In two cases, an axillary fibroadenoma was found, whereas in one case silicone deposits due to implant leakage after breast augmentation were detected. In one biopsy granulomatous inflammatory activity of the LN was detected due to a known sarcoidosis. One patient had previously undergone axillary surgery. These five patients/LNs were subsequently excluded from further analysis. Ninety-five patients were included in the further analysis. The mean age of the patients was 56 ± 16 years. Eighty-two patients received a biopsy of the ipsilateral breast due to a suspicious breast lesion and a biopsy of the axilla. Fifty-five biopsies were performed in the left axilla, and forty in the right axilla (Table 1).

**Table 1:** Description of the study cohort

	Total, (*n* = 95)	Benign, (*n* = 32)	Malignant, (*n* = 63)	*p*-value
Age (years ± SD)	56 ± 15.59	54 ± 14.90	58 ± 15.94	0.12
Simultaneous biopsy of the ipsilateral breast	82	21	61	< 0.001
Side				
Left	55	20	35	
Right	40	12	28	0.52

*SD* standard deviation

### Pathology

Thirty-two (33.7%) biopsies contained healthy LN tissue and sixty-three (66.3%) LN metastases were detected in the remaining 95 patients. Sixty LN metastases were associated with ipsilateral breast cancer. One patient (1.1%) had an LN metastasis consistent with breast cancer but without evidence of a primary site in the breast (cancer of unknown primary, CUP). Two (2.1%) LN metastases were associated with ovarian cancer and head and neck cancer, respectively.

Of all patients, 79 had breast cancer of the ipsilateral breast where the LN biopsy was performed. The pathology including IHC of all patients with breast cancer (*n* = 80, including one patient with CUP) is presented in Table 2.

**Table 2:** Pathology characteristics of patients with current breast cancer of the ipsilateral breast

	Total, (*n* = 80)	Benign, (*n* = 19)	Malignant, (*n* = 61)
Histological subtype			
NST	73	18	55
ILC	4	1	3
Other	3^a^	0	3
Tumor biology			
HR +/HER2 −	55	8	47
HR +/HER2 +	9	4	5
HR −/HER2 +	6	3	3
TNBC	10	4	6
Grading			
1	5	1	4
2	47	10	37
3	28	8	20

*NST* non-special type, *ILC* invasive lobular carcinoma, *HR* hormone receptor, *TNBC* triple-negative breast cancer

^a^ Histological subtypes: two were apocrine and one a combination of NST and ILC

### Conventional B-mode ultrasound

Axillary staging with conventional B-mode ultrasound was performed for all patients.

The mean number of sonographically suspicious LNs were 2.56 ± 1.96 among all patients. Considering the patients in which biopsy proved a benign LN, the mean number of initially sonographically suspicious LNs was 2.09 ± 1.15. In patients with LN metastasis, the mean number of suspicious LNs was 3.03 ± 2.54.

The mean size of the chosen LNs was 12.91 mm ± 4.71 mm × 7.28 mm ± 2.33 mm in benign LNs and 16.67 mm ± 8.10 mm × 10.28 mm ± 5.41 mm in malignant LNs. The mean depth of the chosen LNs was 13.25 mm ± 6.84 mm in benign LNs and 13.81 mm ± 4.32 mm in malignant LNs. There was no statistically significant difference in the depth of the chosen LNs (*p* = 0.62) whereas the size of the LNs was significantly larger in malignant LNs with *p* = 0.017 for the long axis diameter of the ln and *p* < 0.001 for the short axis diameter (Table 3).

**Table 3:** Characteristics of LNs in B-mode ultrasound and velocities measured by SWE (in m/s)

	Total, (*n* = 95)	Benign, (*n* = 32)	Malignant, (*n* = 63)	*p*-value
Number of suspicious LNs^a^ ± SD^b^	2.78 ± 2.31	2.09 ± 1.15	3.10 ± 2.64	0.041
Mean depth of the LN ± SD	13.52 ± 5.39	13.25 ± 6.84	13.81 ± 4.32	0.62
Diameter of the LN in mm				
Long axis (mean ± SD)	15.5 ± 7.24	12.91 ± 4.71	16.67 ± 8.10	0.017
Short axis (mean ± SD)	9.34 ± 4.76	7.28 ± 2.33	10.28 ± 5.41	< 0.001
LN^a^ tissue				
Mean ± SD^b^	3.10 ± 1.44	2.33 ± 0.62	3.48 ± 1.58	< 0.001
Max	10.00	4.97	10.00	–
Min	1.00	1.52	1.00	–
Surrounding tissue				
Mean ± SD	1.67 ± 0.37	1.58 ± 0.32	1.73 ± 0.39	0.06
Max	3.25	2.36	3.25	–
Min	0.98	1.04	0.98	–

^a^ Lymph node

^b^ Standard deviation

### SWE

**Fig. 1:**
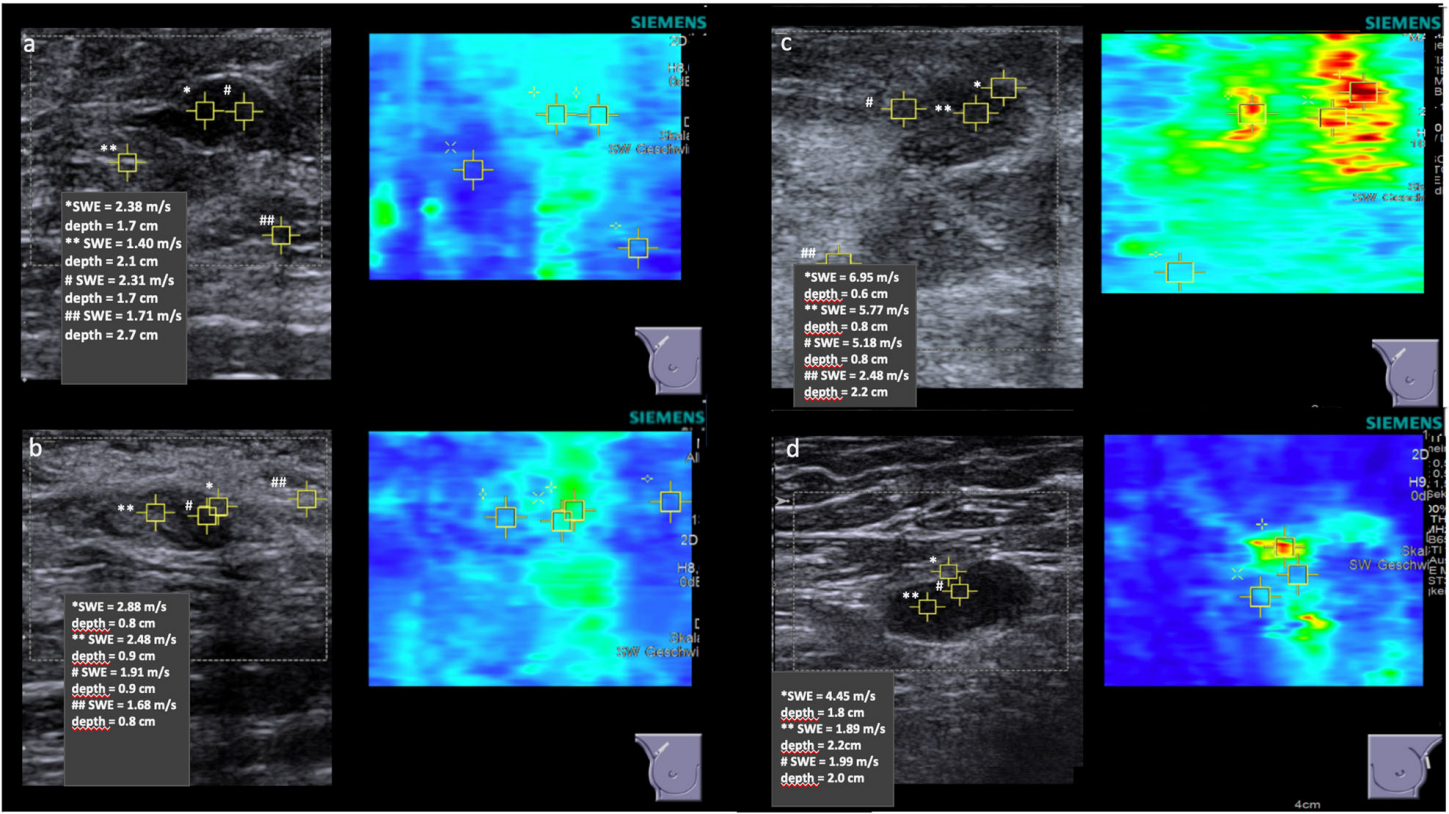
Example measurement of SWE in benign (**a**) and malignant (**b**–**d**) axillary LNs

SWE was performed in all 95 LNs. The region of interest (ROI) was defined as the area with the highest velocity within the respective LN. An example measurement is shown in Fig. 1. Additionally, SWE was measured in the surrounding tissue.

**Fig. 2:**
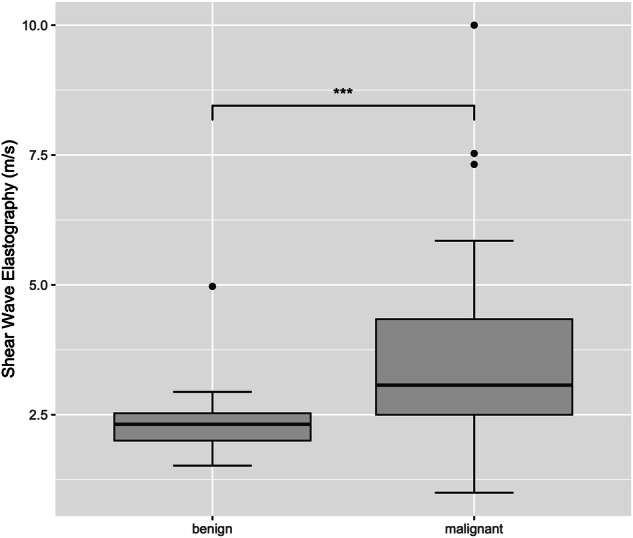
Velocities measured by SWE in benign and malignant LN tissue. *** p < 0.001

Velocities of LN tissue and surrounding tissue showed a mean shear wave velocity of 3.10 ± 1.44 m/s and 1.67 ± 0.37 m/s, respectively. The mean velocity of LN tissue in benign LNs was 2.33 ± 0.62 m/s, while the mean velocity in metastatic LNs was 3.48 ± 1.58 m/s. The velocities of the benign and malignant LNs demonstrate a statistically significant difference (*p* = 0.00015). The mean velocities measured in the surrounding tissue showed no significant difference in both groups. The velocities measured by SWE are presented in Table 3 and Fig. 2.

### ROC analysis

**Fig. 3:**
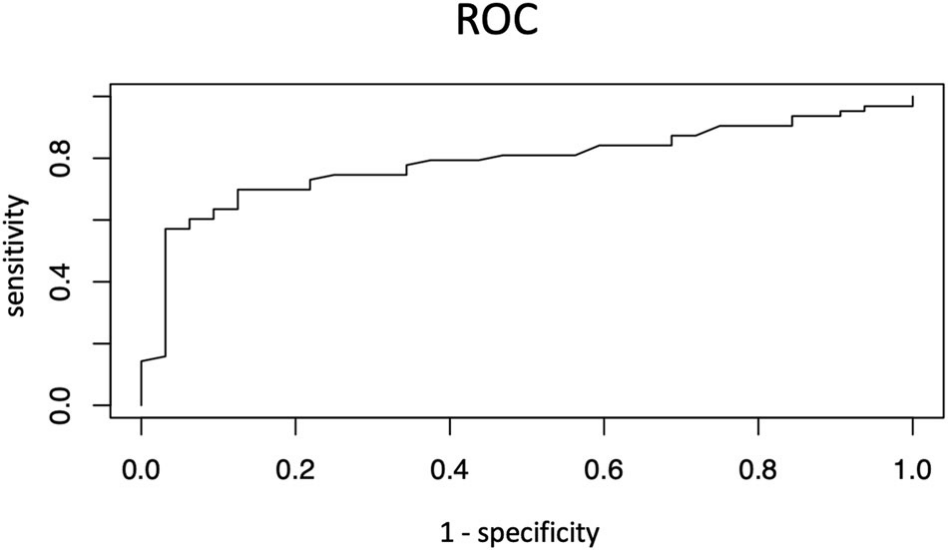
ROC for the velocities measured in LN tissue

AUC for the mean velocity was measured in LN tissue to discriminate between benign and malignant LNs and found to be 0.79 (Fig. 3). The statistically optimal cutoff with the highest Youden Index (0.58) was 2.66 m/s measured in LN tissue. This threshold yielded a sensitivity of 69.8% and a specificity of 87.5%. The corresponding false negative rate stood at 20%, while the false positive rate was 4%.

## Discussion

Ninety-five sonographically suspicious axillary LNs were examined using SWE in a clinically routine setting. The mean velocity measured in LN tissue was 3.10 ± 1.44 m/s for the whole cohort. These values are higher than in sonographically unsuspicious LN, which were collected in a previous study (1.90 ± 0.34 m/s in LN cortex and 2.02 ± 0.37 m/s in LN hilus) [29]. A distinction between the cortex and hilus area of an LN, as performed in the previous study, was not possible in this cohort, as a reliable distinction between these areas was not always possible due to the ultrasound changes in the LNs. In this cohort, LNs with evidence of malignant cells in histopathology had significantly higher mean velocities than benign LNs.

These data are in line with previous literature examining SWE in axillary LNs. Several studies have indicated that velocities measured by SWE correlate with malignancy in axillary LNs. A French study group examined in 2012 eighty-one sentinel LNs using SWE, with seventy LNs being benign and eleven showing metastasis. In their analysis, benign LN showed a significantly lower velocity with 11.32 kPa (converted 1.94 m/s) while LN metastasis showed a higher velocity of 17.47 kPa (converted 2.41 m/s) [36]. Conversion between kPa and m/s was done by using the simplified formula for stiffness in kPa = 3 × (velocity in m/s)^2^ [37, 38]. However, the comparability of these findings to the present study may be limited due to the small number of cases in the metastatic LN group. Bae et al examined SWE on sixty-three LN ex vivo during breast cancer surgery. They demonstrated that LNs later identified as metastatic had a higher mean SWE than benign ones. The mean velocities were 47.7 kPa (converted 3.88 m/s) in metastatic LN and 17.7 kPa (converted 2.43 m/s) in benign ones [39]. The values reported by Bae et al were higher compared to those in the current study; however, direct comparison isn’t feasible as the examination was conducted ex vivo. Several other studies evaluated the use of SWE as part of preoperative axillary staging. In a study by Ng et al qualitative SWE with color patterns had a higher discriminatory power compared with quantitative SWE or B-mode ultrasound [30]. In another analysis conducted by Luo et al, they examined one hundred twenty-one axillary LNs and proposed a cutoff value of 26.90 kPa (equivalent to 3.0 m/s when converted) to distinguish between benign and malignant LNs. It is important to note that this study included patients who underwent both SLNB and ALND. However, there was no guarantee that the LNs measured using SWE corresponded directly to the LNs subsequently operated on. Their findings revealed that the cutoff value of 26.90 kPa (3.0 m/s) achieved a sensitivity of 86.7% and a specificity of 96.7%, both of which were higher than the values reported in the current study [31]. Pulappadi et al additionally demonstrated in a cohort of 48 patients that a velocity measured in the LN cortex higher than 14.9 kPa (converted 2.23 m/s) is associated with malignancy with a sensitivity of 73.7% and a specificity of 81.8% [32]. A further study by Seo et al with fifty-three patients reported a cut-off value of 23.8 kPa (converted 2.82 m/s) to be associated with metastatic LN with a sensitivity of 76.5% and a specificity of 100% [40].

What these discussed studies have in common is the inherent challenge in definitely allocating between LN measured by SWE and those examined pathologically. Pulappadi et al correlated the SWE velocities to the results of the biopsy only, while in the study by Tourasse et al marking on the patients’ skin, as well as the long and short axis diameter of the LN were considered for the paring process [32, 36]. Seo et al used only skin markings for the pairing process [40].

In general, based on the currently available evidence, the threshold velocity warranting a biopsy remains uncertain, given the limited and inconclusive data, ranging between 2.23 m/s and 3.0 m/s, and sensitivity and specificity ranging between 73.7% and 86.7% and 81.8% and 100%, respectively. In this study, a maximum velocity of 2.66 m/s measured in the LN tissue is proposed to adequately differentiate between malignant and benign axillary LN. Within this cut-off velocity, a sensitivity of 69.8% and a specificity of 87.5% can be achieved.

There are several limitations to consider. First, it must be noted that the selection of LNs is solely based on ultrasound criteria. It is further known that non-suspicious LNs may still contain malignant cells as seen in positive SLNBs [41]. It also must be noted that the results of this study only apply to female patients since male patients were excluded from the study cohort. Due to the limited sample size, an analysis of cancer subtypes has not been performed. A final limitation includes the presence of subjective observer bias, which is a common limitation in general ultrasound techniques.

In conclusion, additional SWE provides a quantifiable parameter for axillary staging in breast cancer patients. A recommended cutoff velocity of 2.66 m/s aids in distinguishing between benign and metastatic processes. However, further studies are essential to assess SWE’s efficacy in reducing biopsies in benign axillary LNs and decreasing positive SLNBs due to insufficient preoperative diagnostics.

## Data Availability

The datasets used and analyzed during the current study are available from the corresponding author upon reasonable request.

## Abbreviations

ALND: Axillary lymph node dissection
AUC: Area under the receiver operating characteristic curve
IHC: Immunohistochemistry
LN: Lymph node
ROC: Receiver operating curve
SD: Standard deviation
SLNB: Sentinel lymph node biopsy
SWE: Shear wave elastography
VTIQ: Virtual touch tissue imaging quantification
